# Supplementary material for: The Influence of Colloidal Properties of Carbon Black on Static and Dynamic Mechanical Properties of Natural Rubber
Source: Polymers (Basel). 2022 Mar 16;14(6):1194. doi: 10.3390/polym14061194 (PMC8951755; doi:10.3390/polym14061194)
Supplement: Supplementary file 1 [file polymers-14-01194-s001.zip › polymers-1589724-supplementary-done.pdf]

polymers MDPI

Supplementary Materials

The Influence of Colloidal Properties of Carbon Black on Static and Dynamic Mechanical Properties of Natural Rubber

William Amoako Kyei-Manu [1], Charles R. Herd [2], Mahatab Chowdhury [2], James J. C. Busfield [1,*] and Lewis B. Tunnicliffe [2]

[1] School of Engineering and Materials Science, Queen Mary University of London, London E1 4NS, UK; w.a.kyei-manu@qmul.ac.uk
[2] Birla Carbon, Marietta, GA 30062, USA; charles.herd@adityabirla.com (C.R.H.); mahatab.chowdhury@adityabirla.com (M.C.); lewis.tunnicliffe@adityabirla.com (L.B.T.)
* Correspondence: j.busfield@qmul.ac.uk; Tel.: +44-20-7882-8866

S1. Mixing Procedure of Compounds

Supplementary tables 1-3 show the mixing procedure for the carbon black reinforced natural rubber compounds. The unreinforced counterpart follows a similar mixing procedure with the only exception being no carbon black is added. The mixing process is a three-stage process to ensure uniform dispersion of the CB in the rubber matrix.

Table S1. Stage 1 compounding of natural rubber (NR) compounds.

**Stage 1 : Banbury, 40°C, 77 rpm, 3.0 bar**		
**Time / seconds**	**rpm**	**Operation**
-	77	Load polymer
30	77	Ram down mixing
-	77	Load stearic acid, antiozonant, ZnO, TMQ, Microwax. Add all CB blended, slowly.
15	77	Ram up mixing
60	77	Ram down mixing
-	77	Sweep
90	77	Ram down mixing
-	77	Sweep
90	77	Ram down mixing
-	77	Sweep
90	77	Ram down mixing (150°C max, reduce RPM as necessary)

*Mill 70°C, 25:21 rpm, Gap 0.055-60". Do not cut weight back. Pass through the mill once. Band and cross-blend 6 times. Band 30 seconds, sheet off and let cool for a minimum of 1 hour.

Table S2. Stage 2 compounding of natural rubber (NR) compounds

**Stage 2 : Banbury, 40°C, 77 rpm, 3.0 bar**		
**Time / seconds**	**rpm**	**Operation**
-	77	Load master batch (MB)
120	77	Ram down mixing
~150	77	Discharge

*Mill 70°C, 25:21 rpm, Gap 0.055-60". Pass through the mill once. Band and cross-blend 6 times. Band 30 seconds, sheet off and let cool for a minimum of 1 hour.

Table S3. Stage 3 compounding of natural rubber (NR) compounds

**Stage 3 : Banbury, 25°C, 60 rpm, 3.0 bar**		
**Time / seconds**	**rpm**	**Operation**
-	60	Load half master batch, curatives and then last half of master batch
30	60	Ram down mixing
30	45	Ram down mixing
-	30	Sweep
120	45	Ram down mixing (100°C max, adjust RPM as necessary)
~240	45	Discharge

*Mill 70°C, 25:21 rpm, Gap 0.055-60". Pass through the mill once. Band and cross-blend 6 times. End to end roll 3 times. Band 30 seconds, sheet off and let cool for a minimum of 1 hour before MDR. Let the compound set a minimum of 1 hour before curing.

S2. Transmission Electron Microscopy (TEM) Images of Tested Carbon Blacks

The image below shows the TEM images of the carbon blacks that are tested. The scale bar on each of the images shows a measurement of 100 μm.

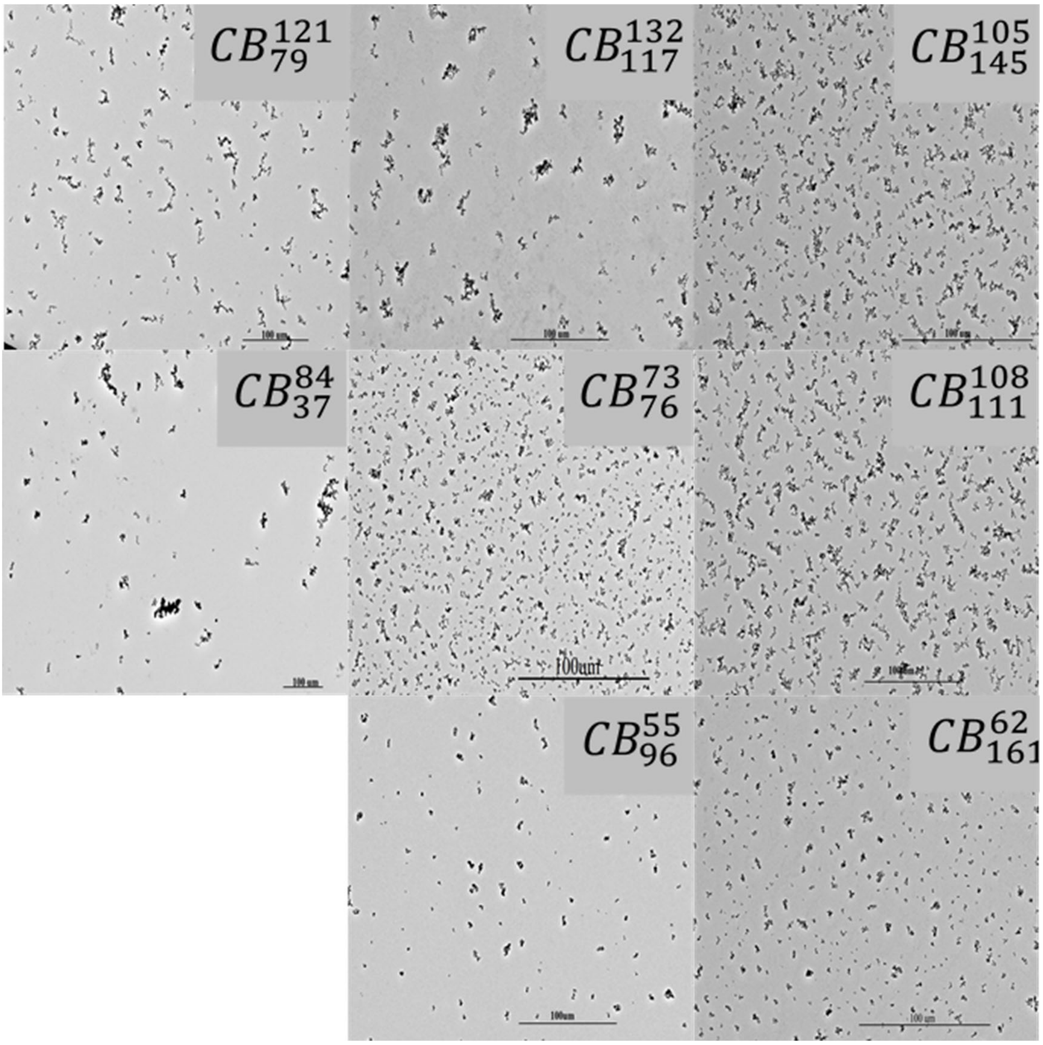

Figure S1. Transmission electron microscopy (TEM) images of tested carbon blacks.

S3. Regression Analyses of Mechanical Hysteresis versus Structure and Surface Area

Cf. Equation 1 of the main manuscript. P values > 0.05 highlighted in red.

Table S4. Multiple regression results of mechanical hysteresis

Percent strain	Cycle Number	C	β_{St}	COAN p	β_{SA}	STSA p	Adjusted R^2
20	1	0.0127	0.0008	0.0014	0.0004	0.0099	0.89
20	2	0.0149	0.0007	0.0011	0.0002	0.0229	0.89
20	3	0.0160	0.0007	0.0005	0.0002	0.0095	0.92
50	1	-0.0036	0.0045	0.0001	0.0004	0.2774	0.95
50	2	0.0057	0.0029	5.04×10^{-5}	0.0005	0.0248	0.96
50	3	0.0154	0.0027	3.14×10^{-5}	0.0005	0.0131	0.97
100	1	-0.1322	0.0147	0.0002	-0.0012	0.2894	0.93
100	2	-0.0051	0.0072	7.31×10^{-5}	-8.14×10^{-5}	0.8578	0.95
100	3	0.0286	0.0063	7.45×10^{-5}	-4.37×10^{-5}	0.9117	0.95
200	1	-1.0534	0.0791	0.0003	-0.0127	0.1055	0.92
200	2	0.0550	0.0227	3.19×10^{-5}	-0.0030	0.0417	0.97
200	3	0.0756	0.0180	2.39×10^{-5}	-0.0020	0.0625	0.97
300	1	-0.2244	0.1264	9.27×10^{-5}	-0.0215	0.0411	0.95
300	2	0.3474	0.0419	6.67×10^{-6}	-0.0062	0.0098	0.98
300	3	0.3614	0.0321	3.29×10^{-5}	-0.0045	0.0385	0.97

S4. Regression Analyses of $|G^*|(\gamma_0)$ versus Structure and Surface Area

Cf. Equation 1 of the main manuscript. P values > 0.05 highlighted in red.

Table S5. Multiple regression results of $|G^*|(\gamma_0)$

γ_0 %	C	β_{St}	COAN p	β_{SA}	STSA p	Adjusted R^2
0.10	0.701	0.0401	0.0018	0.0452	0.0002	0.95
0.16	0.745	0.0388	0.0018	0.0450	0.0002	0.95
0.25	0.825	0.0363	0.0016	0.0441	0.0001	0.96
0.40	0.991	0.0323	0.0014	0.0417	7.10×10^{-5}	0.96
0.62	1.138	0.0282	0.0011	0.0375	4.45×10^{-5}	0.97
0.99	1.310	0.0243	0.0010	0.0314	4.72×10^{-5}	0.97
1.57	1.341	0.0218	0.0012	0.0247	0.0001	0.96
2.49	1.276	0.0204	0.0011	0.0185	0.0003	0.94
3.94	1.179	0.0194	0.0007	0.0131	0.0007	0.94
6.25	1.008	0.0187	0.0002	0.0091	0.0011	0.95
9.90	0.920	0.0176	4.67×10^{-5}	0.0057	0.0016	0.97
15.69	0.884	0.0159	1.24×10^{-5}	0.0032	0.0039	0.98
24.93	0.875	0.0138	7.89×10^{-6}	0.0014	0.0429	0.98
39.51	0.865	0.0119	1.51×10^{-5}	-2.80x10-5	0.9565	0.97
62.62	0.794	0.0112	0.0017	-0.0015	0.2860	0.84

S5. Derivation of Energy Dissipation Density

Equation 7 in the main manuscript can be derived as follows:

The work applied to the specimen in a single sinusoidal cycle is given as the integral of the stress-strain data:

$$W = \int \sigma(t) d\gamma(t) \quad \text{(S1)}$$

The stress response to the applied strain given in terms of linear viscoelastic moduli is:

$$\sigma(t) = \gamma_0[G'(\omega)\sin\omega t + G''(\omega)\cos\omega t] \quad \text{(S2)}$$

The incremental strain is the time derivative of the applied strain cycle:

$$\sigma(t) = \gamma_0[G'(\omega)\sin\omega t + G''(\omega)\cos\omega t] \quad \text{(S3)}$$

Combining Equations S1-S3 gives the applied work in terms of stored and dissipated components. Where the second integral represents the energy dissipated. Note that the notation adopted here is geometry-normalized (i.e. stress/strain vs force/displacement) so we consider the energy dissipated per unit volume of material:

$$W = \gamma_0^2 G'(\omega) \int \sin\omega t \cos\omega t \, d(\omega t) + \gamma_0^2 G''(\omega) \int \cos^2\omega t \, d(\omega t) \quad \text{(S4)}$$

The energy dissipated per unit volume, W_d over a single dynamic cycle is obtained by appropriate integration limits for the second integral in Equation S4:

$$W_d(\omega) = \gamma_0^2 G''(\omega) \int_0^{2\pi} \cos^2\omega t \, d(\omega t) = \pi\gamma_0^2 G''(\omega) \quad \text{(S5)}$$

Equivalent derivations can be performed for stress-controlled deformations and energy density-controlled deformations (Equations 8 and 9 in the main manuscript).

S6. Regression Analyses of $G''(\gamma_0)$ versus Structure and Surface Area

Cf. Equation 1 of the main manuscript. P values > 0.05 highlighted in bold font.

Table S6. Multiple regression results of $G''(\gamma_0)$

γ_0 %	C	β_{St}	COAN p	β_{SA}	STSA p	Adjusted R^2
0.10	-0.0397	0.0019	0.0080	0.004911	1.624×10^{-5}	0.97
0.16	-0.0458	0.0021	0.0059	0.004955	1.800×10^{-5}	0.97
0.25	-0.0564	0.0025	0.0034	0.005039	2.090×10^{-5}	0.97
0.40	-0.0697	0.0031	0.0028	0.005248	3.914×10^{-5}	0.97
0.62	-0.0905	0.0038	0.0031	0.005728	7.389×10^{-5}	0.96
0.99	-0.0704	0.0039	0.0042	0.006082	9.508×10^{-5}	0.95
1.57	-0.0470	0.0037	0.0045	0.006254	7.079×10^{-5}	0.96
2.49	-0.0157	0.0033	0.0049	0.0061	4.840×10^{-5}	0.96
3.94	0.0207	0.0028	0.0090	0.0054	7.800×10^{-5}	0.96
6.25	0.0140	0.0026	0.0119	0.0046	0.0002	0.94
9.90	0.0028	0.0025	0.0045	0.0035	0.0002	0.94
15.69	-0.0021	0.0022	0.0013	0.0025	0.0001	0.96
24.93	-0.0026	0.0018	0.0007	0.0018	0.0001	0.96
39.51	-0.0052	0.0015	0.0005	0.0012	0.0003	0.95
62.62	0.0008	0.0011	0.0046	0.0009	0.0030	0.87

S7. Interpolation of $tan\delta(W_0)$ Data Set

In order to perform regression analysis on the re-scaled $tan\delta$ data (main manuscript Figure 9B), interpolated data of equal spacing in W_0 were generated from the $tan\delta$-strain energy density data sets using the linear interpolation function in Origin 2019B. An example of the interpolated versus experimental data is shown in Figure S1 where interpolated data for three compounds are shown overlaid with the experimental data sets (lines). Agreement between the experimental and the interpolated data sets is excellent.

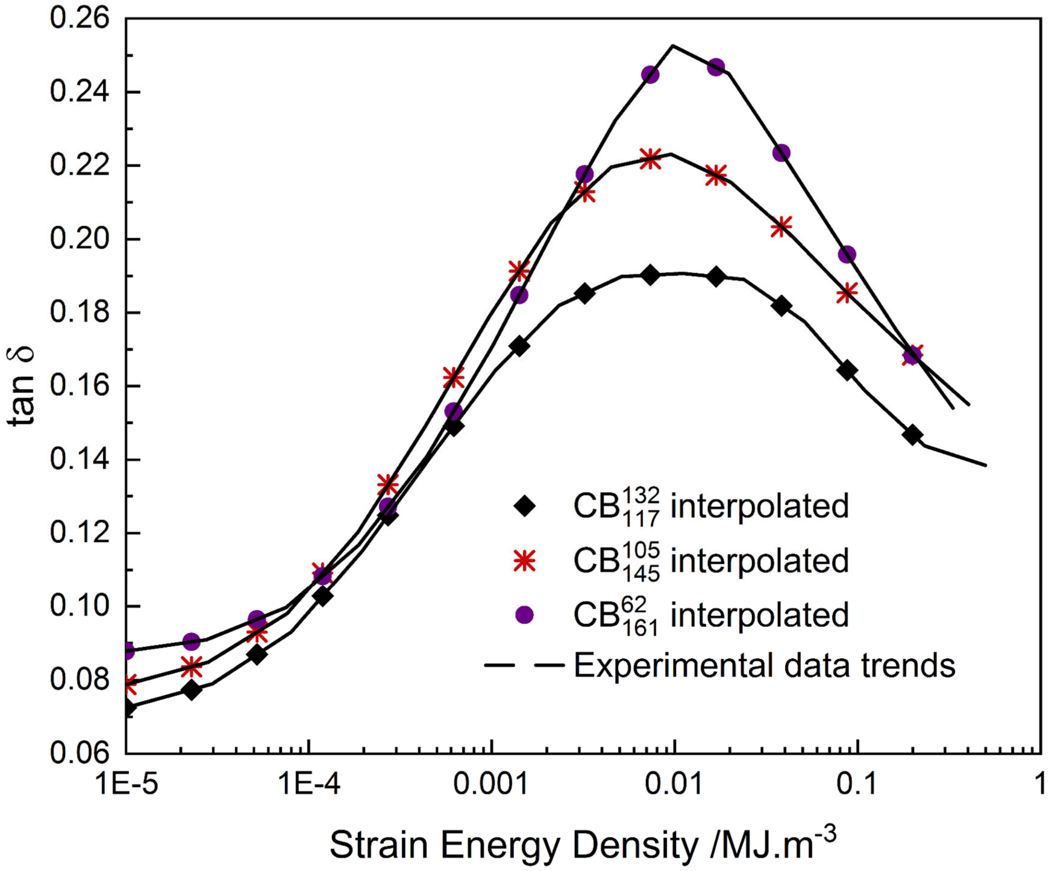

Figure S2. Interpolated $tan\delta(W_0)$ data versus experimental $tan\delta(W_0)$ data for three compounds.

S8. Regression Analyses of $tan\delta(W_0)$ versus Structure and Surface Area

Cf. Equation 1 of the main manuscript. P values > 0.05 highlighted in red.

Table S7. Multiple regression results of $tan\delta(W_0)$

W_0 / MJ.m^{-3}	C	β_{St}	COAN p	β_{SA}	STSA p	Adjusted R^2
0.00001	-0.0001	-0.0001	4.99x10^{-5}	0.0001	1.46x10^{-6}	0.99
2.28x10^{-5}	-9.74x10^{-5}	-9.74x10^{-5}	0.0006	0.0001	3.20x10^{-5}	0.97
5.21x10^{-5}	-7.52x10^{-5}	-7.52x10^{-5}	0.0050	0.0001	0.0002	0.94
0.000119	-5.84x10^{-5}	-5.84x10^{-5}	0.2748	9.95x10^{-5}	0.0283	0.56
0.000271	-5.74x10^{-5}	-5.74x10^{-5}	0.6206	0.00014	0.1156	0.22
0.000619	-7.43x10^{-5}	-7.43x10^{-5}	0.6735	0.00024	0.0867	0.29
0.001413	-0.0001	-0.0001	0.5287	0.0004	0.0320	0.51
0.003224	-0.0002	-0.0002	0.2423	0.0005	0.00430	0.78
0.007356	-0.0003	-0.0003	0.0426	0.0007	0.0001	0.94
0.016788	-0.0002	-0.0002	0.0383	0.0008	1.86x10^{-5}	0.97
0.038312	-7.81x10-5	-7.81x10^{-5}	0.3675	0.0008	2.46x10^{-5}	0.97
0.087431	2.61x10-6	2.61x10^{-6}	0.9776	0.0008	5.56x10^{-5}	0.96
0.199526	8.30x10-5	8.30x10^{-5}	0.3932	0.0007	9.53x10^{-5}	0.95

S9. Interpolation of $J''(\sigma_0)$ Data Set

In order to perform regression analysis on the re-scaled J'' data (main manuscript Figure 10B), interpolated data of equal spacing in σ_0 were generated from the J''-peak dynamic stress data sets using the linear interpolation function in Origin 2019B. An example of the interpolated versus experimental data (symbols) are shown in Figure S2 where

interpolated data for three of the compounds are shown overlaid with the experimental data sets (lines). Agreement between the experimental and the interpolated data sets is excellent.

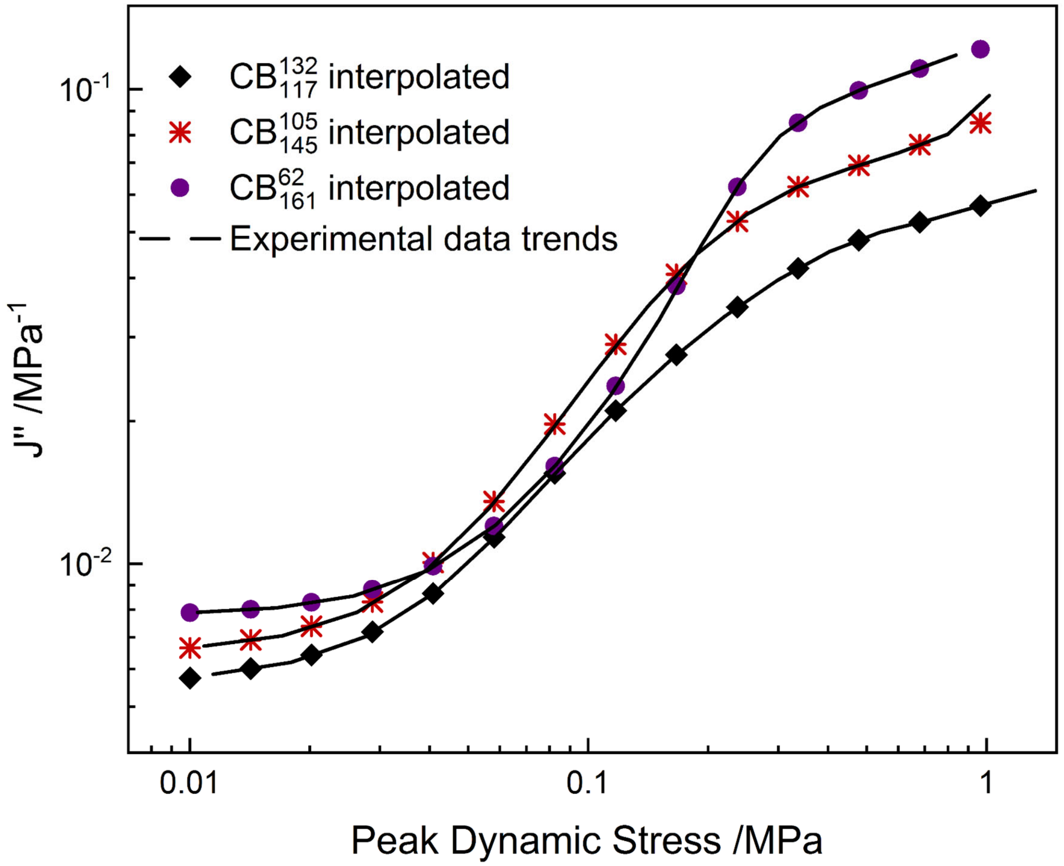

Figure S3. Interpolated $J''(\sigma_0)$ data versus experimental $J''(\sigma_0)$ data for three compounds.

S10. Regression Analyses of $J''(\sigma_0)$ versus Structure and Surface Area

Cf. Equation 1 of the main manuscript. P values > 0.05 highlighted in red.

Table S8. Multiple regression results of $J''(\sigma_0)$

σ_0 / MPa	C x10³	β_{St}x10³	COAN p	β_{SA}x10³	STSA p	Adjusted R²
0.010	16.785	-0.0513	0.0051	-0.0369	0.0041	0.86
0.014	18.687	-0.0551	0.0120	-0.0468	0.0050	0.83
0.020	21.830	-0.0624	0.0178	-0.0618	0.0040	0.83
0.029	26.590	-0.0730	0.0138	-0.0829	0.0017	0.87
0.041	33.728	-0.0912	0.0031	-0.1094	0.0002	0.94
0.058	43.664	-0.1220	0.0015	-0.1354	0.0002	0.95
0.082	57.031	-0.1737	0.0123	-0.1552	0.0041	0.84
0.117	72.659	-0.2474	0.0237	-0.1571	0.0308	0.70
0.167	86.578	-0.3419	0.0130	-0.1093	0.1386	0.68
0.237	93.416	-0.4443	0.0015	0.0150	0.7700	0.84
0.336	92.608	-0.5152	0.0001	0.1622	0.0051	0.95
0.478	88.245	-0.5454	5.69x10^{-5}	0.2752	0.0002	0.97
0.680	85.009	-0.5710	1.68x10^{-5}	0.3647	2.37x10^{-5}	0.99
0.966	80.989	-0.5860	5.96x10^{-6}	0.45648	3.15x10^{-6}	0.99
